# Pre-treatment radiological factors associated with poor functional outcome in an Asian cohort of large vessel occlusion acute ischemic stroke patients undergoing mechanical thrombectomy

**DOI:** 10.3389/fneur.2024.1415233

**Published:** 2024-06-26

**Authors:** Joshua Y. P. Yeo, Kevin Soon Hwee Teo, En Ying Tan, Clyve Yaow, H. Hariz, H. S. Lim, B. J. M. Ng, Y. H. L. Wong, Cantiriga Subramaniam, Andrew Makmur, Weiping Han, Mark Y. Y. Chan, Ching-Hui Sia, Mingxue Jing, Benjamin Y. Q. Tan, David K. K. Tang, Leonard Leong Litt Yeo

**Affiliations:** ^1^Department of Medicine, National University Hospital, Singapore, Singapore; ^2^Division of Neurology, Department of Medicine, National University Hospital, Singapore, Singapore; ^3^Yong Loo Lin School of Medicine, National University of Singapore, Singapore, Singapore; ^4^Department of Diagnostic Imaging, National University Hospital, Singapore, Singapore; ^5^Institute of Molecular and Cell Biology, Singapore, Singapore; ^6^National University Heart Centre, National University Hospital, Singapore, Singapore

**Keywords:** stroke, acute ischemic stroke, prognosis, symptomatic intracranial haemorrhage, endovascular thrombectomy

## Abstract

**Background and aims:**

Endovascular thrombectomy (EVT) is the current standard of care for large vessel occlusion (LVO) acute ischemic stroke (AIS); however, up to two-thirds of EVT patients have poor functional outcomes despite successful reperfusion. Many radiological markers have been studied as predictive biomarkers for patient outcomes in AIS. This study seeks to determine which clinico-radiological factors are associated with outcomes of interest to aid selection of patients for EVT for LVO AIS.

**Methods:**

A retrospective study of patients who underwent EVT from 2016 to 2020 was performed. Data on various radiological variables, such as anatomical parameters, clot characteristics, collateral status, and infarct size, were collected alongside traditional demographic and clinical variables. Univariate and multivariate analysis was performed for the primary outcomes of functional independence at 3 months post-stroke (modified Rankin Scale 0–2) and secondary outcomes of in-hospital mortality and symptomatic intracranial hemorrhage.

**Results:**

The study cohort comprised 325 consecutive patients with anterior circulation LVO AIS (54.5% male) with a median age of 68 years (interquartile range 57–76). The median NIHSS was 19. Age, hypertension, hyperlipidaemia, National Institutes of Health Stroke Scale (NIHSS), Alberta mCTA score, ASPECTS, clot length, thrombus HU and mTICI score and the angle between ICA and CCA were associated with functional outcomes at 3 months on univariate analysis. On multivariate analysis, age, Alberta mCTA collaterals and NIHSS were significantly associated with functional outcomes, while ASPECTS approached significance.

**Conclusion:**

Among the many proposed radiological markers for patients in the hyperacute setting undergoing EVT, the existing well-validated clinico-radiological measures remain strongly associated with functional status.

## Highlights

- **What is already known on this topic:** There are numerous proposed radiological markers related to outcomes for mechanical thrombectomy in the literature such as clot length, surface phenotype and vascular tortuosity.
- **What this study adds:** In our cohort, these signs were not significantly associated with functional outcomes or risk of procedural complications. Existing well-validated signs remain the most closely associated with post-procedural outcomes.
- **How this study might affect research, practice or policy:** Amidst the widening indications for endovascular thrombectomy in acute ischaemic stroke with large vessel occlusion, patient selection should be guided by existing well-validated markers.

## Introduction

Endovascular thrombectomy (EVT) has emerged as the standard of care for patients with large vessel occlusion (LVO) acute ischemic stroke (AIS), which has led to its increasing adoption in stroke centers worldwide. When treated within 24 h of symptom onset, endovascular thrombectomy increases patients’ chances of survival and good functional outcomes (1).

However, approximately 10–20% of patients who undergo EVT do not achieve successful recanalization (2), and up to two-thirds of patients who undergo EVT do not achieve functional independence despite successful recanalization (3). Furthermore, the procedure itself is not without risk; complications of EVT include access site complications, device-related complications, arterial perforation, dissection, and intracranial hemorrhage (4). It is therefore germane to pre-procedurally identify clinico-radiological factors that may portend a more favorable outcome.

Radiological signs that are readily available on a patient’s index CT scan have generated interest as predictors for patients undergoing EVT for LVO AIS. For example, poor baseline collateral flow status and Alberta Stroke Program Early CT Score (ASPECTS), assessed via CT angiography, are associated with a larger ischemic core and worse functional outcomes (5, 6). Other radiological variables which have been investigated include clot characteristics including clot length, density, surface phenotype, truncal versus branch-type occlusions and the presence of a meniscus sign (7–11). Further radiological variables that have been studied pertain to vascular anatomy and include parameters that quantify vascular tortuosity such as the aortic arch type (12, 13).

In this study, we sought to determine associations between clinico-radiological factors and outcomes of interest in LVO AIS to determine which factors have the best predictive capability in guiding patient selection for EVT.

## Methods

### Study design

This was an observational cohort study of consecutive patients who underwent EVT for LVO AIS from a single comprehensive stroke center over a five-year period from 2016 to 2020. Patients underwent non-enhanced CT and high-resolution CT angiogram (CTA) as part of their hyperacute stroke assessment. The CT scans were performed on a 128-slice multidetector helical scanner (Philips Inc.) with a 60-70-mL bolus injection of iohexol contrast. Scan parameters were: source axial thickness of 0.625 mm, matrix 512 × 512, and 120 kV. The scan coverage was from the aortic arch to the vertex, and the source images were reformatted into 10×2.5 mm axial, coronal, and sagittal maximum intensity projection images. The initiation of the multiphasic CTA (mCTA) was triggered by the technician upon the initial visibility of contrast in the aortic arch. Subsequently, CTA images were captured with a 2 mm slice thickness, starting from the aortic arch, proceeding through the circle of Willis, and extending to the vertex. Axial, coronal, and sagittal projections were obtained. The pre-treatment mCTA included immediate (peak arterial), first (peak venous), and second (late venous) phases. The delayed scans occurred 10 and 12 s later.

All patients above the age of 18 who underwent EVT for ischaemic stroke secondary to large vessel occlusion identified on multidetector CT angiogram (CTA) were included. The selection criteria for patients for patients undergoing EVT were in accordance with the American Heart Association/American Stroke Association guidelines for the early management of patients with AIS (14). Patients that had hemorrhagic stroke at presentation or patients who were ultimately deemed not to have LVO AIS were excluded. Patients who did not have an adequate field of imaging to include the aortic arch to the vertex or had poor quality imaging were also excluded.

The study was approved by an institutional ethics committee and research board (NHG Domain Specific Review Board Reference Number 2022/00109).

### Data collection

Clinical variables including age, sex, comorbidities, mRS score, NIHSS, and tPA administration, as well as data relevant to the EVT procedure - onset-to-puncture time, thrombolysis in cerebral infarction (TICI) score, thrombus characteristics (irregular surface, presence of meniscus sign or calcified) - were collected from patients’ electronic medical records. Radiological variables collected included: ASPECTS, clot length, thrombus Hounsfield Units, aortic arch type, truncal occlusion, meniscus sign, irregular clot surface, angle of internal carotid artery (ICA) and common carotid artery (CCA), and Alberta multiphase CT angiography (mCTA) score. Aortic arch type was defined using Madhwal’s classification (15). Truncal occlusions were defined as large vessel occlusions where all major branches and bifurcation sites were clearly visible beyond the occlusion segment. The meniscus sign was defined as a clot with a concave appearance on angiography. The data were collected independently by 2 residents; differences between the datasets were resolved by a senior consultant neurologist.

### Outcome measures

The primary outcome measure was functional independence (FI), defined as a Modified Rankin Scale (mRS) score of 0–2 at 3 months post-stroke. Secondary outcome measures were in-hospital mortality and symptomatic intracranial hemorrhage (sICH), as defined by ECASS2 consensus criteria (16). Other procedural complications that were observed included groin hematomas and distal emboli.

### Statistical analyses

Clinically relevant variables were incorporated into the analysis. Normally distributed continuous variables were expressed as mean ± standard deviation (SD), while categorical variables were presented as percentages. We employed the Pearson χ2 test (or Fisher exact test when applicable) for categorical variables and Student’s t-test for normally distributed continuous variables. Univariate regression was performed to identify significant covariates with the primary and secondary outcomes.

Multivariate binary logistic regression models were constructed using baseline covariates with statistically significant (*p* < 0.05) associations to identify independent predictors of the primary and secondary outcomes. Adjusted odds ratios (aORs) with corresponding 95% confidence intervals (CIs) and *p*-values were calculated for all statistical analyses.

Statistical analyses were conducted using IBM SPSS Statistics version 26. A statistically significant finding was indicated by a two-sided *p* value of <0.05.

## Results

**Figure 1:**
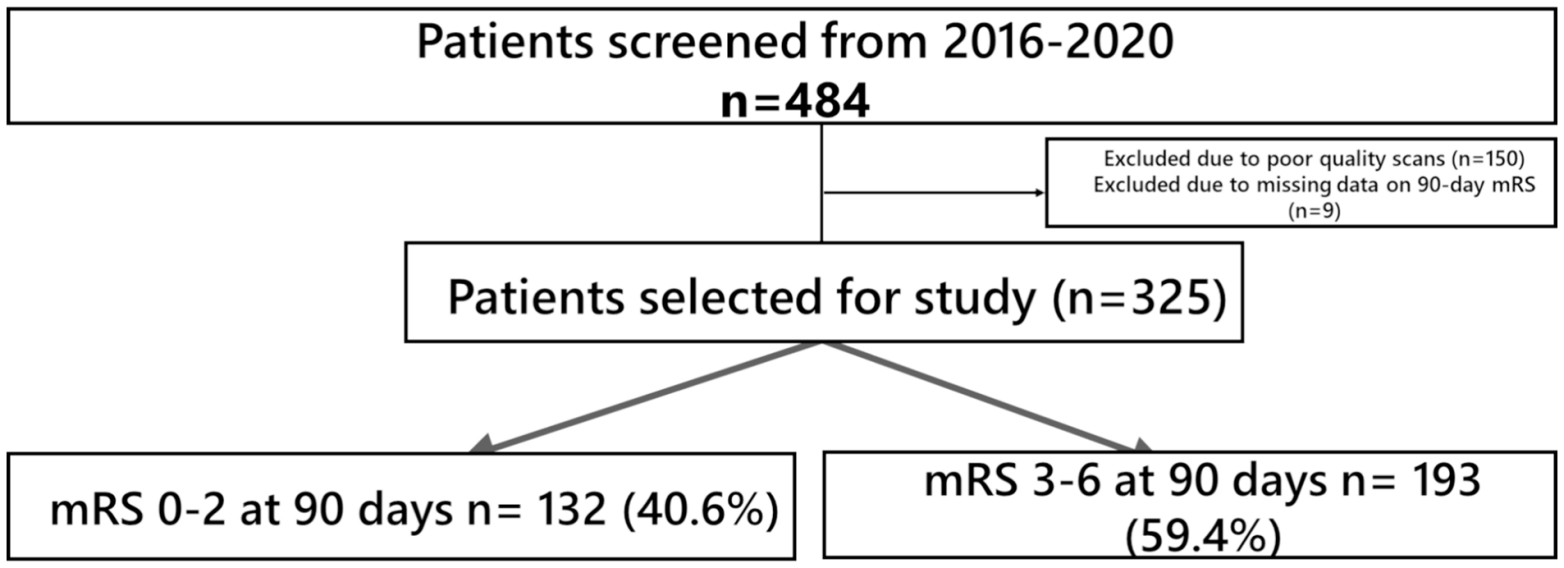
Patient selection.

The demographic and clinical characteristics of the study cohort are shown in Table 1. A total of 484 patients (45.5% women) of median age 68 that underwent EVT for LVO AIS were included. 150 patients were excluded due to factors such as poor-quality scans or insufficient coverage up to the aortic arch. (Figure 1) while 9 patients had missing 90-day mRS data with a cohort of 325 for analysis. 132 patients (42.2%) attained FI at 3 months post-stroke. In hospital mortality was 10.2%, and 32 (9.6%) patients developed sICH post-EVT.

**Table 1:** Baseline characteristics of the study cohort (*n* = 325).

		FI	No FI
n	325	132	193
Age, median [IQR]	68 [57–76]	63 [53–70.3]	72 [63–79]
Race			
Chinese	220 (65.9)	88 (66.7)	127 (65.8)
Indian	29 (8.7)	29 (22.0)	42 (21.8)
Malay	71 (21.3)	9 (6.8)	18 (9.3)
Others	14 (4.2)	6 (4.5)	6 (3.1)
Sex (female)	152 (45.5)	56 (42.4)	92 (47.6)
Hypertension	239 (71.6)	83 (63.0)	150 (77.7)
Diabetes mellitus	94 (28.1)	26 (19.7)	67 (34.7)
Hyperlipidemia	185 (55.4)	64 (48.5)	117 (60.6)
Ischemic heart disease	76 (22.8)	26 (19.7)	50 (25.9)
Congestive cardiac failure	41 (12.3)	14 (10.6)	27 (14.0)
Smoking	63 (18.9)	29 (22.5)	33 (17.8)
Atrial fibrillation	164 (49.1)	59 (45.0)	101 (52.3)
Previous stroke	49 (14.5)	18 (13.6)	31 (16.0)
SBP on arrival (mmHg), median [IQR]	149 [133–168]	148 [131–165]	149 [135–168]
DBP on arrival (mmHg), median [IQR]	82 [71–95]	82 [71.5–91.5]	82 [70.5–96.5]
NIHSS, median [IQR]	19 [10–27]	15 [11–20]	20 [17–23]
Thrombolysis with rTPA	215 (64.4)	84 (64.1)	126 (65.3)
Onset to puncture time (mean, mins)	272.2	273.0	273.4
TOAST classification*			
Large artery atherosclerosis	99	41	58
Cardioembolic	180	65	115
Other determined cause	3	1	2
Cryptogenic	46	25	21
Occlusion site			
ICA (including terminal ICA)	88	22	64
MCA (M1, M2)	218	102	116
ACA (A1)	2	1	1
Tandem occlusion	26	8	17
Baseline mRS			
0	279	125	147
1	13	5	8
2	14	2	11
mRS at 3 months post-stroke			
0	55	53	-
1	40	39	-
2	41	40	-
3	54	-	52
4	78	-	73
5	25	-	24
6	45	-	44
In-hospital mortality	35 (10.5)	-	35
Symptomatic intracranial hemorrhage	32 (9.6)	2	29

ACA, anterior cerebral artery; ICA, internal carotid artery; IQR, interquartile range; MCA, middle cerebral artery; mRS, modified Rankin scale; NIHSS, National Institutes of Health Stroke Scale; rTPA, recombinant tissue plasminogen activator; SBP, systolic blood pressure; DBP, diastolic blood pressure. Categorical variables presented as *n* (%) unless otherwise stated. *The TOAST classification denotes five subtypes of ischemic stroke: (1) large-artery atherosclerosis, (2) cardioembolic, (3) small-vessel occlusion, (4) stroke of other determined etiology, and (5) stroke of undetermined etiology.

On univariate analysis, the radiological variables which were associated with FI at 3 months post-stroke were Alberta mCTA score, ASPECTS, clot length, thrombus Hounsfield Units (HU), angle between ICA and CCA, as well as the modified TICI score (Table 2). On multivariate analysis, the radiological variables that were significantly associated with FI was the Alberta mCTA score (OR 13.4 95% CI 1.39–130, *p* = 0.025), with the ASPECTS approaching significance (OR 0.693, 95% CI 0.473–1.01, *p* = 0.059).

**Table 2:** Analysis of variables associated with 90-day functional independence.

		Univariate analysis				Multivariate analysis	
		FI	No FI	*p*-value	OR (95%CI)	Adjusted OR (95% CI)	*p*-value
Age (mean)		61.2	69.5	**<0.001**	**1.05 (1.03–1.07)**	1.056 (1.00–1.11)	**0.036**
Female		56 (42.4%)	92 (47.6%)	0.366			
Race							
Chinese		88	127	0.793			
Malay		29	42				
Indian		9	18				
Others		6	6				
Clinical factors							
Hypertension		83 (62.95%)	150 (77.7%)	**0.004**	**2.06 (1.26–3.36)**	0.983 (0.237–4.079)	0.981
Diabetes Mellitus		26 (19.7%)	67 (34.7%)	**0.004**	**2.17 (1.29–3.65)**	4.23 (1.02–17.5)	**0.047**
Hyperlipidemia		64 (48.5%)	117 (60.6%)	**0.032**	**1.64 (1.05–2.56)**	1.62 (0.483–5.40)	0.436
Smoking		29 (22.5)	33 (17.8%)	0.317			
Ischaemic heart disease		26 (19.7%)	50 (25.9%)	0.23			
Congestive cardiac failure		14 (11.6%)	27 (15.0%)	0.494			
Pre-admission mRS 0–2		132 (100%)	166 (86.0%)	**<0.001**	**N.A.**	32.5 *10^7	0.999
TOAST	Large artery atherosclerosis	41 (32.0%)	56 (28.9%)	0.152			
	Cardioembolic	61 (47.6%)	115 (59.3%)				
	Small vessel disease	0	0				
	Other determined cause	1 (0.78%)	2 (1.03%)				
	Cryptogenic	25 (19.5%)	21 (10.82%)				
Prior stroke		17 (12.9%)	31 (16.2%)	0.500			
Atrial fibrillation		59 (45.0%)	101 (52.3%)	0.214			
Systolic Blood Pressure on arrival (mean)		149	152	0.297			
Diastolic Blood Pressure on arrival (mean)		84.3	86.1	0.408			
Thrombolysis with rTPA		84 (64.1%)	126 (65.3%)	0.906			
Onset-to-puncture (mean, mins)		283.7	280.3	0.88			
NIHSS (median)		15	20	**<0.001**	**1.08 (1.04–1.13)**	1.12 (1.02–1.24)	**0.021**
Radiological factors							
Alberta mCTA <3		86 (95.6%)	79 (70.5%)	**<0.001**	**8.98 (3.05–26.5)**	13.1 (1.35–127)	**0.026**
MCA Top-to-bottom distance (mean, cm)		0.689	0.704	0.627			
Aortic arch type (median)		2	2	0.871			
Angle between ICA and CCA (mean)		34	38	**0.039**	**1.01 (1.00–1.03)**	0.99 (0.96–1.03)	0.78
Meniscus sign present		35 (30.7%)	60 (36.6%)	0.368			
Irregular surface of clot		43 (37.7%)	47 (28.5%)	0.119			
Occlusion location:		43 (62.3%)	78 (66.7%)	0.633			
Truncal							
Bifurcation		26 (37.7%)	39 (33.3%)				
Clot burden score (median)		4	4	0.174			
ASPECTS (median)		9	7	**<0.001**	**0.709 (0.603–0.833)**	0.69 (0.47–1.00)	0.052
MCA-hyperdensity		70 (59.3%)	118 (67.4%)	0.173			
Clot length (mean, cm)		1.26	1.54	**0.014**	**1.71 (1.11–2.66)**	1.01 (0.43–2.35)	0.981
Thrombus HU (non-contrasted CT)		117 (39.1)	170 (41.6)	**0.04**	**1.03 (1.00–1.05)**	1.05 (0.99–1.12)	0.126
TICI 2B/3		116 (92.1%)	137 (71.7%)	**< 0.001**	**0.219 (0.107–0.449)**	0.50 (0.065–3.76)	0.50

*P*-values < 0.05 are bolded.

**Figure 2:**
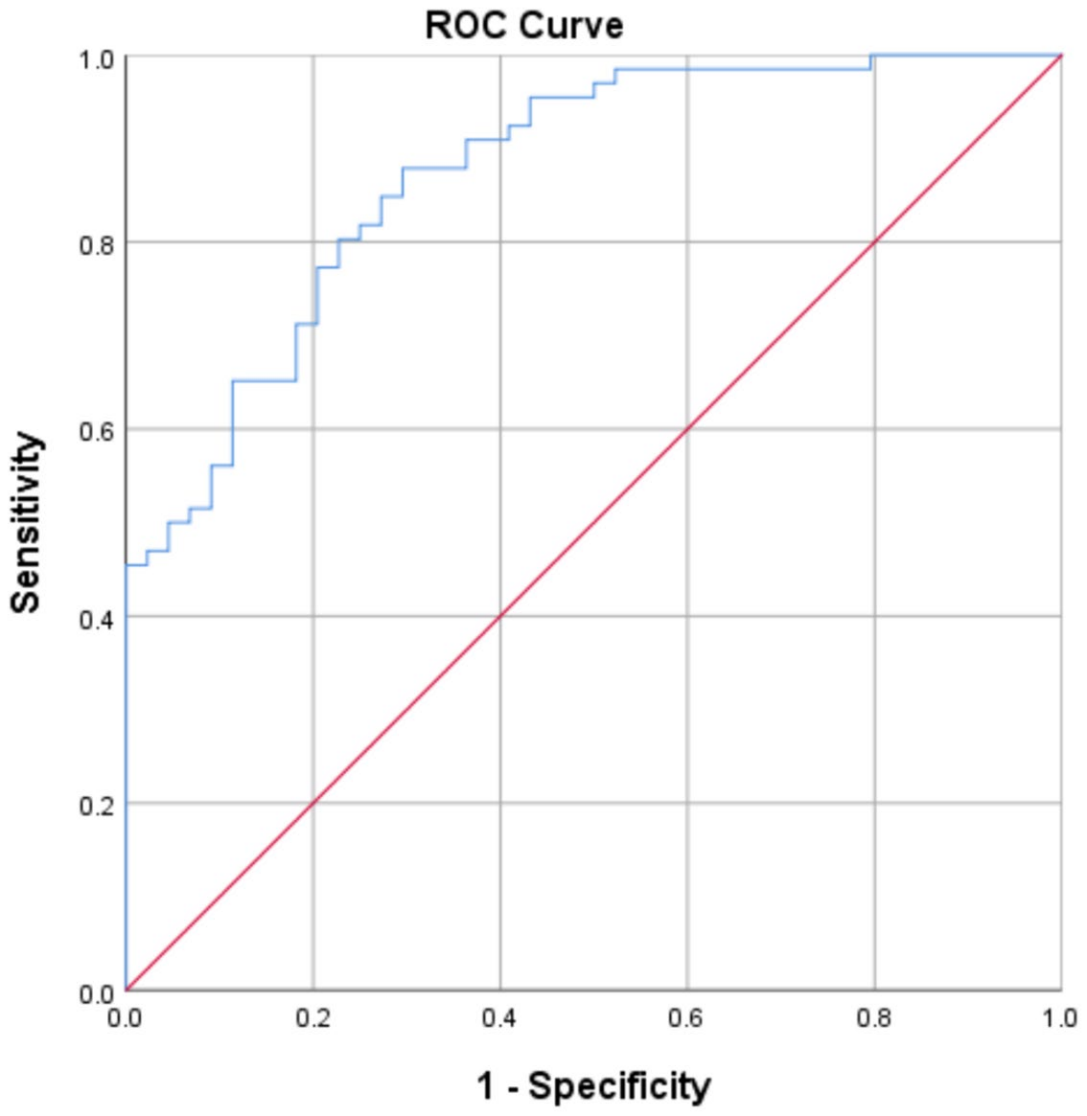
ROC curve for the logistic regression model.

**Figure 3:**
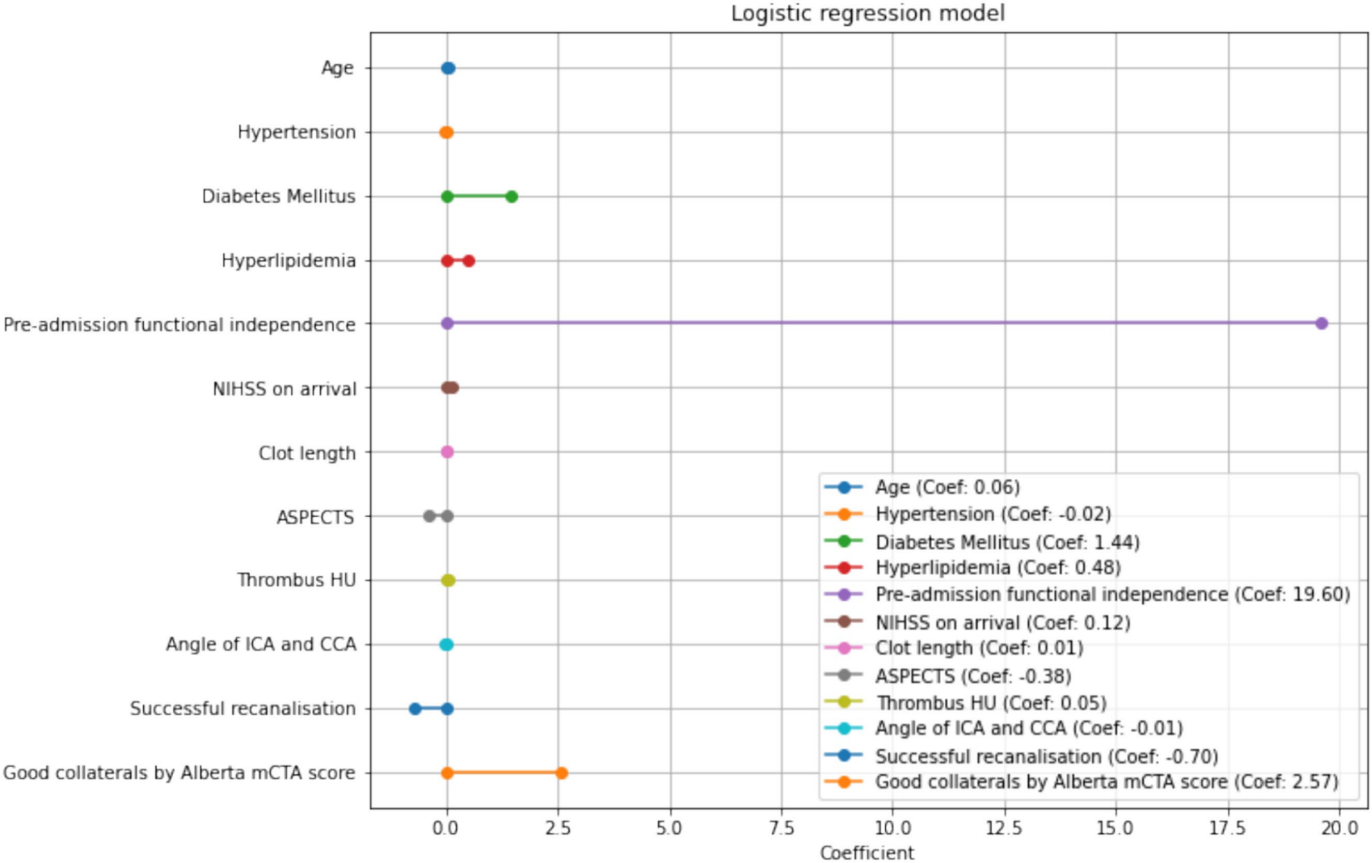
Coefficients in the logistic regression model.

The clinical variables significantly associated with FI in the multivariate analysis was younger age (OR 1.062, 95% CI 1.01–1.12, *p* = 0.021), the presence of diabetes (OR 4.14 95%CI 1.01–16.9, *p* = 0.048), and higher NIHSS (OR 1.13 95%CI 1.02–1.25, *p* = 0.0160). This model demonstrated an AUROC 0.87 (Figure 2) with a Brier’s score of 0.14. The absolute magnitude of their coefficients in the logistic regression is shown in Figure 3.

For the radiological variables, Alberta mCTA score, aortic arch type, and TICI recanalisation status were associated with in-hospital mortality on univariate analysis. ASPECTS remained significantly associated with in-hospital mortality post EVT on multivariate analysis (OR 0.720 95%CI 0.522–0.993, *p* = 0.045) (Supplementary Table S1). The model had a Brier’s score of 0.060 and an AUROC of 0.816 (Supplementary Figure S1).

ASPECTS and clot length were significantly associated with the development of SICH post-EVT on univariate and multivariate analyses. Favorable aortic arch types also showed a trend to significance with SICH (OR 0.433 95% CI 0.186–1.007, *p* = 0.052) on multivariate analysis (Supplementary Table S2). The model had a Brier’s score of 0.077 and an AUROC of 0.82 (Supplementary Figure S2).

## Discussion

In this cohort of patients, the radiological variables which were associated with FI at 3 months post-EVT were the Alberta mCTA score, ASPECTS, clot length, thrombus Hounsfield Units (HU), angle between ICA and CCA, as well as the achievement of first-pass TICI 2B or 3 recanalisation. On multivariate analysis, only the Alberta mCTA score was significantly associated with FI, with the ASPECTS approaching significance. With respect to secondary outcomes, the ASPECTS was significantly associated with the development of post-EVT SICH on both univariate and multivariate analyses. Thrombus clot length was also found to be predictive of post-EVT SICH on multivariate analysis.

The most widely-studied radiological markers that predict endovascular or functional outcomes in stroke include the ASPECTS (17) or multiphasic CT-angiogram score (18). In this study cohort, patients who achieved FI at 3 months post-EVT had a higher median ASPECTS and higher percentages of possessing a good collateral circulation as compared to patients who did not attain FI. Both mCTA collateral status and ASPECTS remained associated with FI post-EVT on multivariable analysis. A lower ASPECTS was also associated with the development of SICH. Our study validates findings from previous studies done in Western cohorts which have identified the ASPECTS and mCTA as prognostic neuroimaging markers for functional outcomes (6, 18). Other studies have also shown the association between a low ASPECTS score as a predictor of post-EVT SICH (19). In particular, newer, more recent trials have attempted to demonstrate the efficacy of EVT in large ischemic-core volume infarcts with an ASPECTS score of 3–5 or a core volume of greater than 50 mL on perfusion imaging, with a tendency towards greater rates of functional independence (20).

Radiological clot characteristics (clot length, density, truncal type occlusions and surface characteristics) have also generated interest as potential biomarkers that may predict functional outcomes in EVT and LVO AIS. For example, thrombus lengths greater than 8 mm were associated with failure of recanalization following intravenous thrombolysis (21), and shorter thrombus lengths were found to be associated with better functional outcomes following EVT for LVO AIS (22). In our study, clot length was associated with post-EVT SICH. Clot density was previously identified to be predictive of successful recanalization on EVT (23, 24). However, our study corroborates other studies in the literature which did not identify a significant association between clot density and better outcomes post-EVT (25).

Truncal-type occlusions have been regarded as a surrogate marker for LVO from intracranial atherosclerosis (11) and were found to be less amenable to mechanical thrombectomy using a stent-retriever approach (26) but the presence of the sign was not reported to be predictive of the recanalization rate or clinical outcomes post-EVT (27). In this study population, there was no significant association identified between truncal-type occlusions and the outcomes of interest.

Other clot-related signs that may aid in prognosticating post-EVT outcomes is the meniscus sign (28). It has been postulated that clots with a meniscus sign may be rich in red blood cells and break down easily compared to a fibrin clot. Supporting this are studies which identified a higher recanalization rate and better functional outcomes in patients who received direct aspiration as opposed to stent retriever for LVO AIS presenting with a meniscus sign (10). However, a more recent multicentric study involving prospective local registries of high-volume centres subsequently demonstrated little prognostic significance for the meniscus sign, consistent with what was found in our center (26). One possible explanation is that modern neurointerventional techniques apply a combined approach utilizing both stenting and direct aspiration, negating the individual effects of either approach. Furthermore, new thrombectomy devices are becoming more effective as the technology progresses and are able to handle a wider variety of occlusive clots that were previously refractory to earlier techniques.

Further radiological variables that have been studied relate to vascular anatomy and the technical aspects of performing mechanical thrombectomy. For instance, Shirakawa et al. (29) found that the MCA tortuosity, measured using the top-bottom distance of the proximal M1 segment on angiography, was significantly associated with the incidence of post-EVT hemorrhage. Carotid artery tortuosity measured using the angle between the CCA and ICA was previously found to be an independent predictor of achieving first-pass recanalization in endovascular thrombectomy (30). In this study, carotid artery tortuosity was associated with functional independence at 3 months post-EVT on univariate analysis and the aortic arch type was associated with in-hospital mortality and SICH post-EVT.

### Limitations

The limitations of this study include recruitment of the cohort from a single stroke center. While reflective of a heterogenous Southeast Asian population, the results of this study may not be fully generalizable to other populations. Distinct risk factor profiles have emerged from population studies, with Asians displaying a higher susceptibility to intracranial stenosis, while Caucasians exhibit a higher prevalence of atrial fibrillation or extracranial stenosis (31). This study also focused on patients with anterior circulation occlusions, and the findings may not be generalizable to patients who present with posterior circulation LVO. Finally, our cohort is derived from a single study and is of a moderate size. It may not be adequately powered to perform subgroup analysis. Future studies involving multi-center collaboration may yet identify other prognostic radiological biomarkers within patient subgroups.

A further limitation arises from incomplete data owing to re-identification losses and data cleaning. To avoid introducing any biases into the multivariate model, imputation was not performed. As a result, not all of the patients were included in the analysis, and may limit the value of the model as a predictor. Despite this limitation, the mean squared difference between the predicted probabilities and actual was 0.14 with a good AUROC of 0.87. Future studies with larger cohorts would help to ascertain the generalizability of our findings.

Our study shows that despite the interest in intra-and pre-procedural angiographic signs, well-validated clinico-radiological variables remain the most valuable in terms of prognostic value. In particular, older age, NIHSS, diabetes mellitus and the Alberta mCTA score was associated with functional independence while thrombus clot length was associated with SICH. Clinicians may choose to review these markers in assessing the likelihood of a patient to benefit from mechanical thrombectomy for large vessel occlusion strokes.

## Conclusion

Among the proposed radiological markers for patients in the hyperacute setting, existing well-validated clinico-radiological measures such as the ASPECTS and Alberta mCTA grading remain strongly associated with functional status. Thrombus clot length demonstrates an association with functional status and risk of SICH post-EVT. Future studies involving multi-center collaboration may yet identify other prognostic radiological biomarkers within patient subgroups.

## Data availability statement

The original contributions presented in the study are included in the article/Supplementary material, further inquiries can be directed to the corresponding author.

## Ethics statement

The studies involving humans were approved by NHG Domain Specific Review Board. The studies were conducted in accordance with the local legislation and institutional requirements. Written informed consent for participation was not required from the participants or the participants' legal guardians/next of kin in accordance with the national legislation and institutional requirements.

## Author contributions

JY: Writing – original draft, Writing – review & editing. KT: Writing – original draft, Writing – review & editing. ET: Validation, Writing – review & editing. CY: Data curation, Writing – review & editing. HH: Data curation, Writing – review & editing. HL: Data curation, Writing – review & editing. BN: Data curation, Writing – review & editing. YW: Data curation, Writing – review & editing. CS: Data curation, Writing – review & editing. AM: Data curation, Writing – review & editing. WH: Writing – review & editing. MC: Writing – review & editing. C-HS: Writing – review & editing. MJ: Methodology, Supervision, Writing – review & editing. BT: Resources, Supervision, Writing – review & editing. DT: Resources, Supervision, Writing – review & editing. LY: Formal analysis, Methodology, Resources, Supervision, Writing – review & editing.

## Supplementary material

SUPPLEMENTARY FIGURE 1 ROC curve for prediction of SICH.

SUPPLEMENTARY FIGURE 2 ROC curve for prediction of mortality.
