# Supplementary figures and images for: Pre-treatment radiological factors associated with poor functional outcome in an Asian cohort of large vessel occlusion acute ischemic stroke patients undergoing mechanical thrombectomy
Source: Front Neurol. 2024 Jun 26;15:1415233. doi: 10.3389/fneur.2024.1415233 (PMC11234891; doi:10.3389/fneur.2024.1415233)

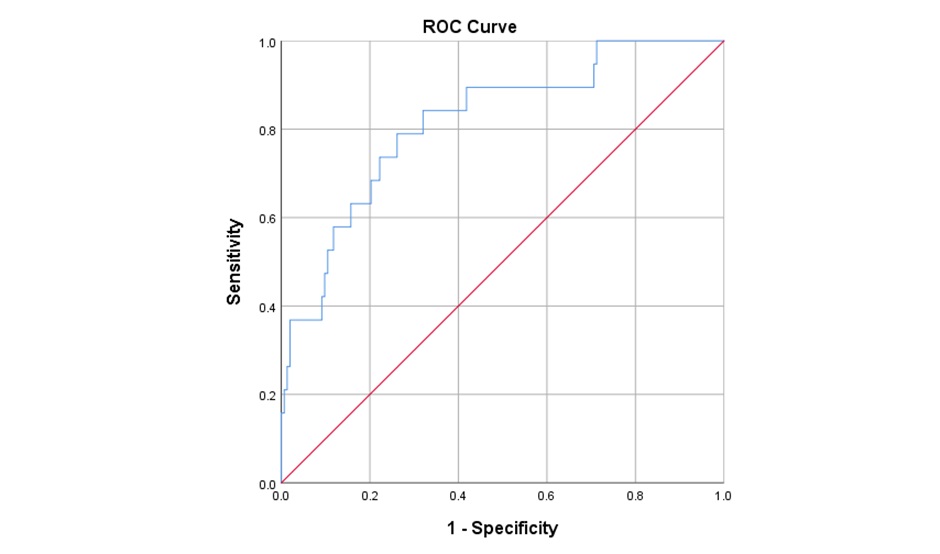

Supplement: SUPPLEMENTARY FIGURE 1 — ROC curve for prediction of SICH. [file Image_1.JPEG]

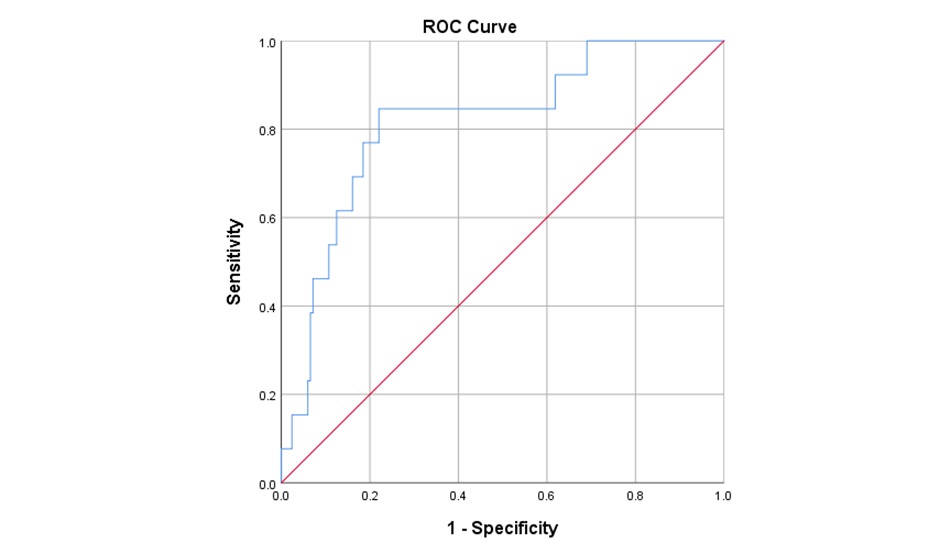

Supplement: SUPPLEMENTARY FIGURE 2 — ROC curve for prediction of mortality. [file Image_2.JPEG]
