# Supplementary material for: Pre-treatment radiological factors associated with poor functional outcome in an Asian cohort of large vessel occlusion acute ischemic stroke patients undergoing mechanical thrombectomy
Source: Front Neurol. 2024 Jun 26;15:1415233. doi: 10.3389/fneur.2024.1415233 (PMC11234891; doi:10.3389/fneur.2024.1415233)
Supplement: Supplementary file 3 [file Table_1.docx]

Supplemental Table 1: Analysis of variables associated with inpatient mortality

|  | | Univariate Analysis | | | | Multivariate Analysis | |
| --- | --- | --- | --- | --- | --- | --- | --- |
|  | | Mortality | Survived | P-value | OR (95% CI) | Adjusted OR(95% CI) | P-value |
| Age (mean) | | 69.97 | 65.48 | 0.054 |  |  |  |
| Female | | 12(40.0%) | 140(46.1%) | 0.569 |  |  |  |
| Race | Chinese | 19 | 201 | 0.874 |  |  |  |
|  | Malay | 8 | 63 |  |  |  |  |
|  | Indian | 2 | 27 |  |  |  |  |
|  | Others | 1 | 13 |  |  |  |  |
| Hypertension | | 23 (76.7%) | 216(71.1%) | 0.672 |  |  |  |
| Diabetes Mellitus | | 13(43.3%) | 81(26.6%) | 0.058 |  |  |  |
| Hyperlipidemia | | 19 (63.3%) | 166 (54.6%) | 0.443 |  |  |  |
| Smoking | | 5 (17.9%) | 58 (19.7%) | 1.000 |  |  |  |
| Ischaemic heart disease | | 12 (40.0%) | 64 (21.1%) | **0.023** | **2.45 (1.12 - 5.35)** | 0.67 (0.14-3.15) | 0.62 |
| Congestive cardiac failure | | 5 (17.9%) | 36 (12.8%) | 0.395 |  |  |  |
| TOAST | Large artery atherosclerosis | 13 (38.2%) | 84 (28.8%) | 0.378 |  |  |  |
|  | Cardioembolic | 19 (55.9%) | 161 (55.1%) |  |  |  |  |
|  | Small vessel disease | 0 | 0 |  |  |  |  |
|  | Other determined cause | 0 | 3 (1.03%) |  |  |  |  |
|  | Cryptogenic | 2 (5.88%) | 44 (15.1% |  |  |  |  |
| Prior stroke | | 7 (25.0%) | 41 (14.2%) | 0.513 |  |  |  |
| Atrial fibrillation | | 15 (50.0%) | 149 (49.2%) | 1.000 |  |  |  |
| Systolic Blood Pressure on arrival (mean) | | 149.50 | 151.92 | 0.623 |  |  |  |
| Diastolic Blood Pressure on arrival (mean) | | 87.33 | 85.20 | 0.540 |  |  |  |
| Pre-admission mRS 0-2 | | 24 (80.0%) | 282 (92.8%) | **0.029** | **3.15(1.17 - 8.51)** | 2.87 (0.46 - 18.1) | 0.26 |
| IV TPA | | 22 (73.3%) | 193 (63.7%) | 0.325 |  |  |  |
| Onset-to-puncture(mean,mins) | | 241.79 | 286.40 | 0.063 |  |  |  |
| Alberta mCTA<3 | | 6 (46.2%) | 31 (15.8%) | **0.014** | **4.79 (1.50 - 15.3)** | 3.2 (0.908-11.3) | 0.070 |
| MCA Top-to-bottom distance(mean,cm) | | 0.773 | 0.686 | 0.163 |  |  |  |
| Aortic arch type (median) | | 1 | 2 | **0.043** | **0.56 (0.32 - 1.00)** | 0.64 (0.26-1.58) | 0.34 |
| Angle between ICA and CCA (mean) | | 37.0 | 37.0 | 0.997 |  |  |  |
| Meniscus sign present | | 9 (34.6%) | 89 (34.2%) | 1.000 |  |  |  |
| Irregular surface of clot | | 10 (38.5%) | 80 (30.7%) | 0.506 |  |  |  |
| Occlusion location:  Truncal  Bifurcation | | 15 (83.3%) | 109 (63.0%) | 0.119 |  |  |  |
|  |  | 3 (16.7%) | 64 (37.0%) |  |  |  |  |
| Clot burden score (median) | | 4 | 4 | 0.445 |  |  |  |
| NIHSS (median) | | 19 | 20 | **0.008** | **1.04 (1.00 - 1.07)** | 1.05 (0.95 - 1.17) | 0.34 |
| ASPECTS (median) | | 7 | 9 | **0.001** | **0.645 (0.527 - 0.789)** | 0.76 (0.55 - 1.05) | 0.091 |
| MCA-hyperdensity | | 21 (72.4%) | 172 (63.2%) | 0.417 |  |  |  |
| Clot length (mean,cm) | | 1.62 | 1.39 | 0.148 |  |  |  |
| Thrombus HU (non-contrasted CT) | | 42.52 | 40.37 | 0.327 |  |  |  |
| TICI2B/3 | | 18 (60.0%) | 243 (82.4%) | **0.007** | **0.32 (0.15 - 0.71)** | 0.30 (0.08 - 1.09) | 0.067 |
