# Supplementary material for: Pre-treatment radiological factors associated with poor functional outcome in an Asian cohort of large vessel occlusion acute ischemic stroke patients undergoing mechanical thrombectomy
Source: Front Neurol. 2024 Jun 26;15:1415233. doi: 10.3389/fneur.2024.1415233 (PMC11234891; doi:10.3389/fneur.2024.1415233)
Supplement: Supplementary file 4 [file Table_2.docx]

Supplemental Table 2: Analysis of variables associated with symptomatic intracranial haemorrhage

|  | | Univariate Analysis | | | | Multivariate Analysis | |
| --- | --- | --- | --- | --- | --- | --- | --- |
|  | | Present | Absent | P-value | OR (95% CI) | Adjusted OR(95% CI) | P-value |
| Age (mean) | | 65.7 | 66.0 | 0.910 |  |  |  |
| Female | | 15 (46.9%) | 137 (45.5%) | 1.00 |  |  |  |
| Race | Chinese | 19 | 201 | 0.411 |  |  |  |
|  | Malay | 6 | 65 |  |  |  |  |
|  | Indian | 5 | 23 |  |  |  |  |
|  | Others | 2 | 12 |  |  |  |  |
| Hypertension | | 25 (78.1%) | 213 (70.8%) | 0.537 |  |  |  |
| Diabetes Mellitus | | 9 (28.1%) | 84 (27.9%) | 1.000 |  |  |  |
| Hyperlipidemia | | 19 (59.4%) | 165 (54.8%) | 0.710 |  |  |  |
| Smoking | | 7 (22.6%) | 56 (19.2%) | 0.637 |  |  |  |
| Ischaemic heart disease | | 8 (25.0%) | 68 (22.6%) | 0.825 |  |  |  |
| Congestive cardiac failure | | 5 (16.7%) | 35 (12.6%) | 0.566 |  |  |  |
| TOAST | Large artery atherosclerosis | 13 (30.9%) | 83 (28.52%) | 0.474 |  |  |  |
|  | Cardioembolic | 16 (38.1%) | 162 (55.67%) |  |  |  |  |
|  | Small vessel disease | 0 | 0 |  |  |  |  |
|  | Other determined cause | 0 | 3 (1.03%) |  |  |  |  |
|  | Cryptogenic | 3(7.14%) | 43 (14.78%) |  |  |  |  |
| Prior stroke | | 3 (9.68%) | 28 (90.3%) | 0.543 |  |  |  |
| Atrial fibrillation | | 16 (50.0%) | 148 (49.3%) | 1.000 |  |  |  |
| Systolic Blood Pressure on arrival (mean) | | 160 | 151 | 0.08 |  |  |  |
| Diastolic Blood Pressure on arrival (mean) | | 93.1 | 84.4 | **0.026** | **1.02 (1.01 - 1.04)** | 1.022 (.994-1.051) | 0.123 |
| Pre-admission mRS 0-2 | | 2 (6.3%) | 26 (8.6%) | 1.000 |  |  |  |
| IV TPA | | 24 (75.0%) | 191 (63.7%) | 0.245 |  |  |  |
| Onset-to-puncture (mean,mins) | | 274 | 283 | 0.784 |  |  |  |
| Alberta mCTA<3 | | 7 (31.8%) | 30 (16.1%) | 0.080 |  |  |  |
| MCA Top-to-bottom distance (mean,cm) | | 0.694 | 0.693 | 0.992 |  |  |  |
| Aortic arch type (mean rank) | | 2 | 2 | **0.010** | **0.448 (0.241-0.833)** | 0.433 (0.186 - 1.007) | **0.052** |
| Angle between ICA and CCA (mean) | | 38.5 | 36.9 | 0.706 |  |  |  |
| Meniscus sign present | | 8 (28.6%) | 89 (34.6%) | 0.675 |  |  |  |
| Irregular surface of clot | | 11 (39.3%) | 79 (30.6%) | 0.393 |  |  |  |
| Occlusion location:  Truncal  Bifurcation | | 14 (63.6%) | 110 (65.5%) | 1.000 |  |  |  |
|  |  | 8 (36.4%) | 58 (34.5%) |  |  |  |  |
| Clot burden score(median) | | 4 | 4 | 0.603 |  |  |  |
| NIHSS (median) | | 21 | 19 | **0.036** | **1.03 (0.990-1.06)** | 0.981 (0.894 - 1.077) | 0.689 |
| ASPECTS (median) | | 6 | 9 | **0.000** | **0.641 (0.526 - 0.781)** | 0.598 (0.449 - 0.796) | **<0.001** |
| MCA-hyperdensity | | 24 (80.0%) | 168 (62.2%) | 0.070 |  |  |  |
| Clot length (mean,cm) | | 1.77 | 1.36 | **0.013** | **1.88 (1.12-3.15)** | 2.136 (1.062 - 4.296) | **0.033** |
| Thrombus HU (non-contrasted CT) | | 43.6 | 40.2 | 0.103 |  |  |  |
| TICI2B/3 | | 22 (68.8%) | 238 (81.5%) | 0.101 |  |  |  |
